# Supplementary material for: Effect of Psilocybin and Ketamine on Brain Neurotransmitters, Glutamate Receptors, DNA and Rat Behavior
Source: Int J Mol Sci. 2022 Jun 16;23(12):6713. doi: 10.3390/ijms23126713 (PMC9224489; doi:10.3390/ijms23126713)
Supplement: Supplementary file 1 [file ijms-23-06713-s001.zip › ijms-1769269SM.pdf]

International Journal of
Molecular Sciences

MDPI

Supplementary Material S1

Original western blot details

Examples of the whole immunoblots with the same time exposure to all analyzed proteins.

Each part of blots is separately presented in Fig.3 (B, C, E, F) with time exposure specific for each protein.

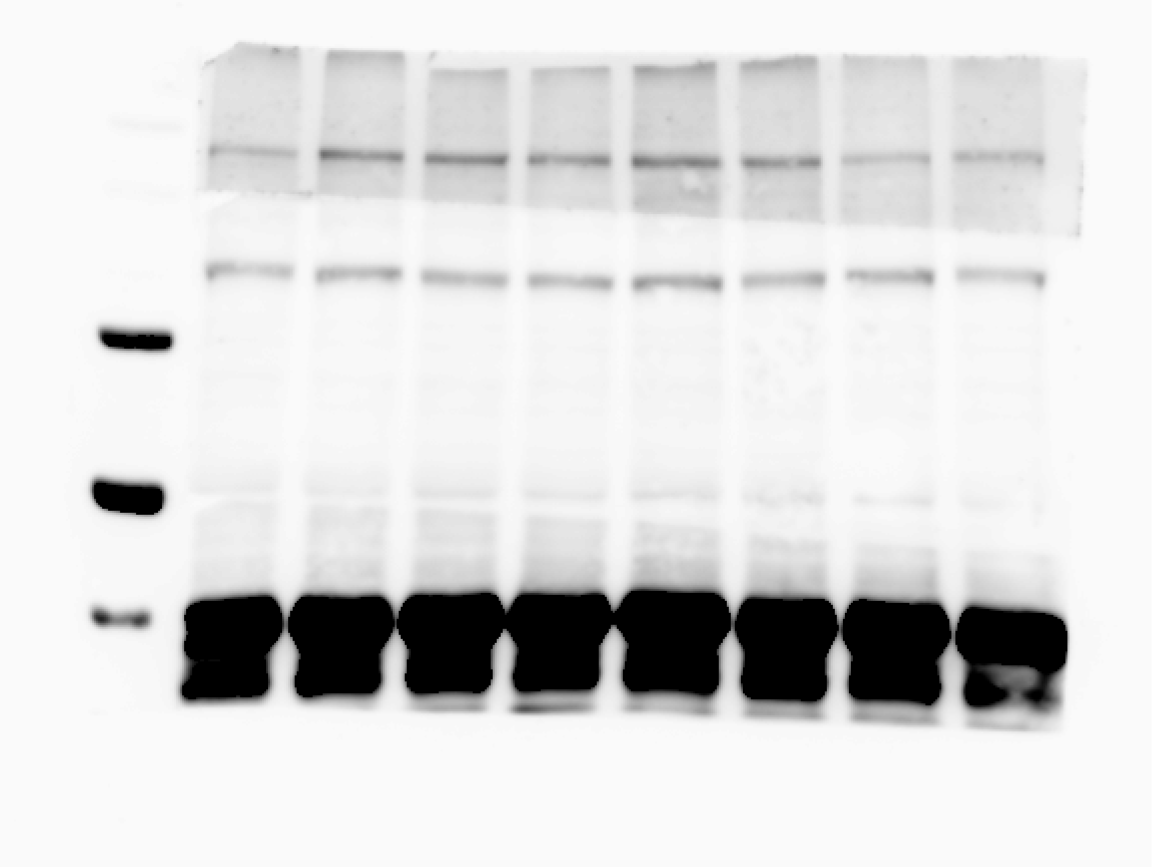

Figure S1.1. : The uncropped, untouched, full original image of western blot presenting GluN2B, GluA1 and GAPDH.

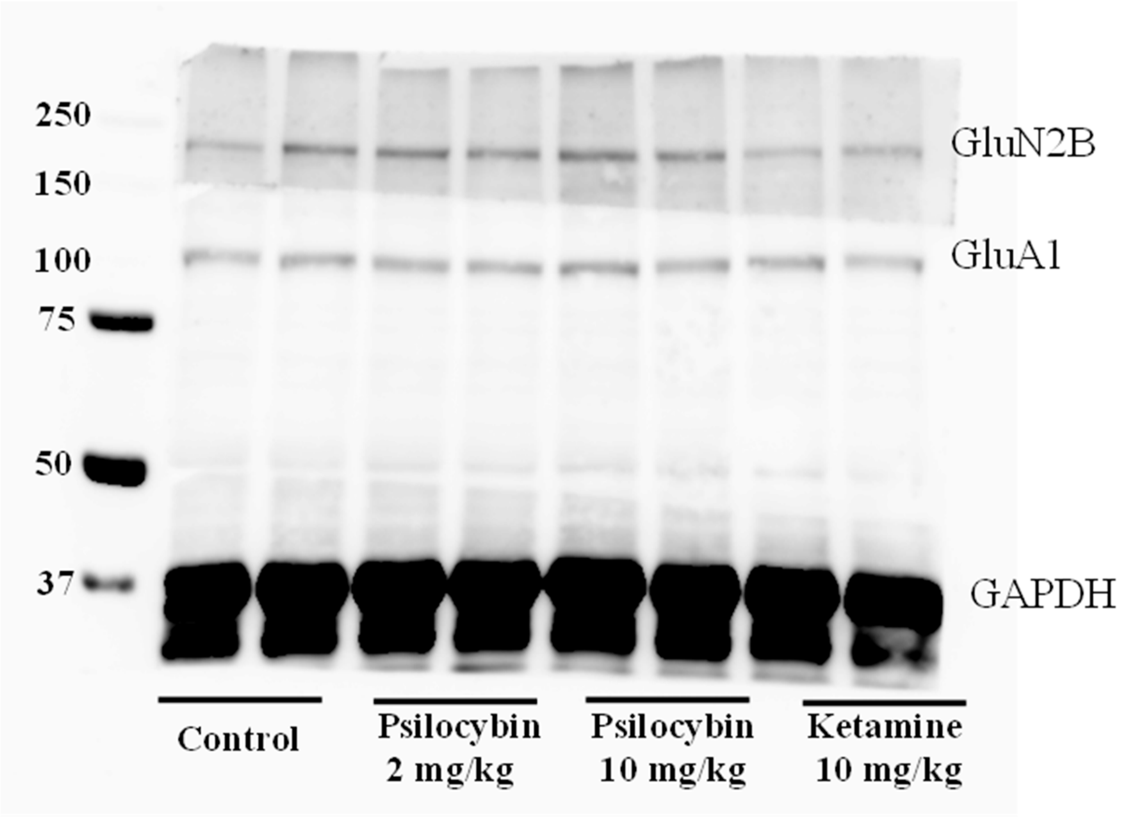

Figure S1.2. : The full image of western blot with marked GluN2B, GluA1 and GAPDH, groups and ladder.

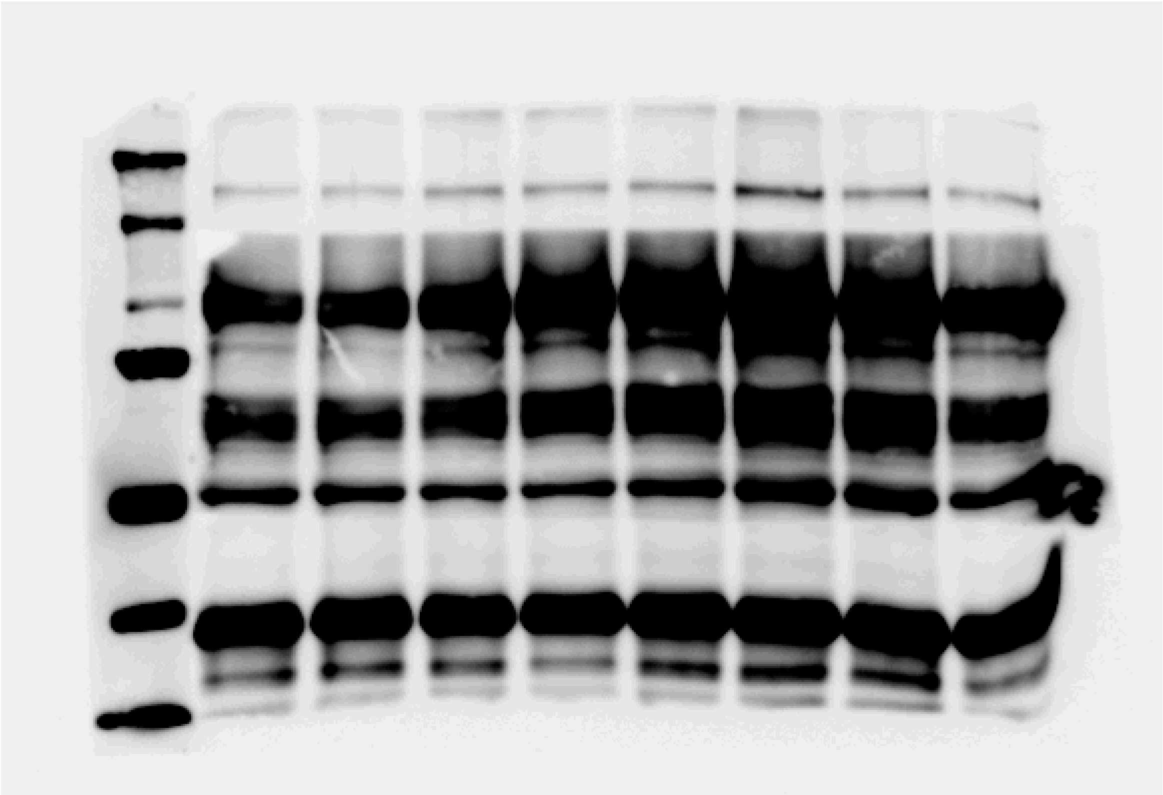

Figure S1.3.: The uncropped, untouched, full original image of western blot presenting GluN2A, GluA2 and GAPDH.

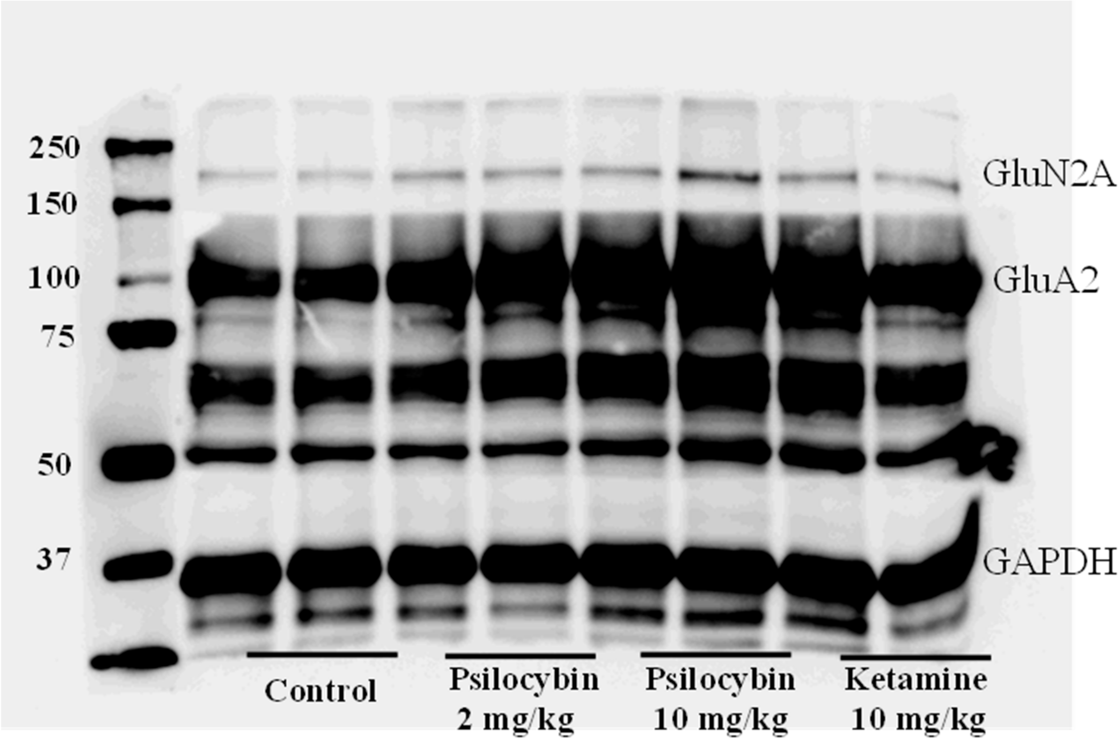

Figure S1.4. : The full image of western blot with marked GluN2A, GluA2 and GAPDH, groups and ladder.

Supplementary Material S2

Histological tracing of microdialysis probes in frontal cortex and thalamus.

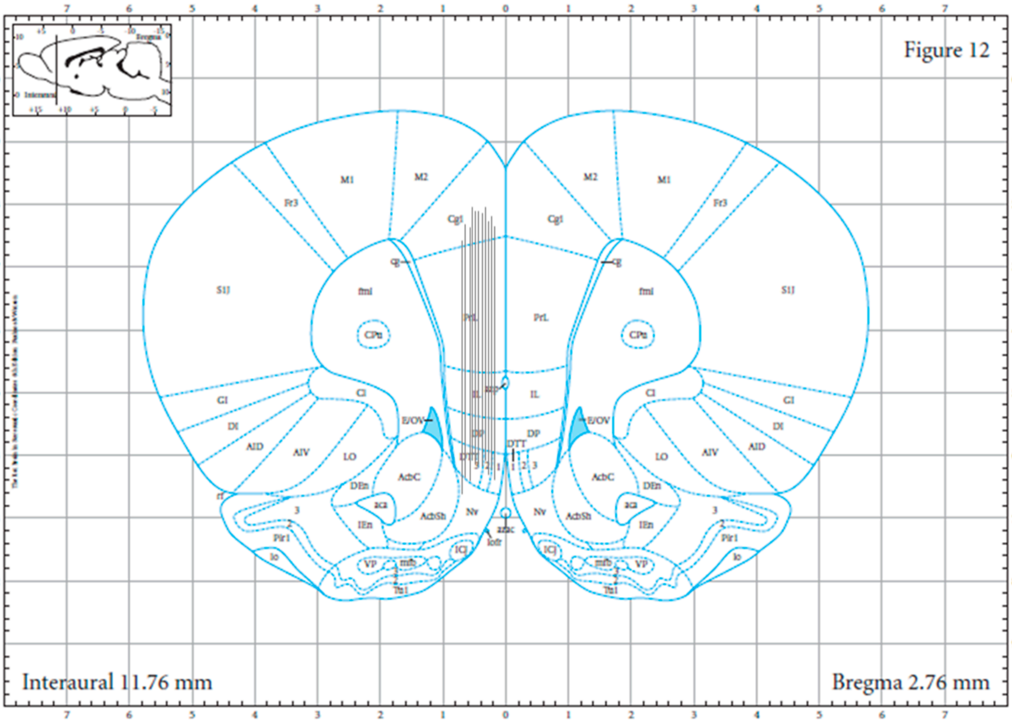

Figure S2.1.: Histological tracing of microdialysis probes in frontal cortex

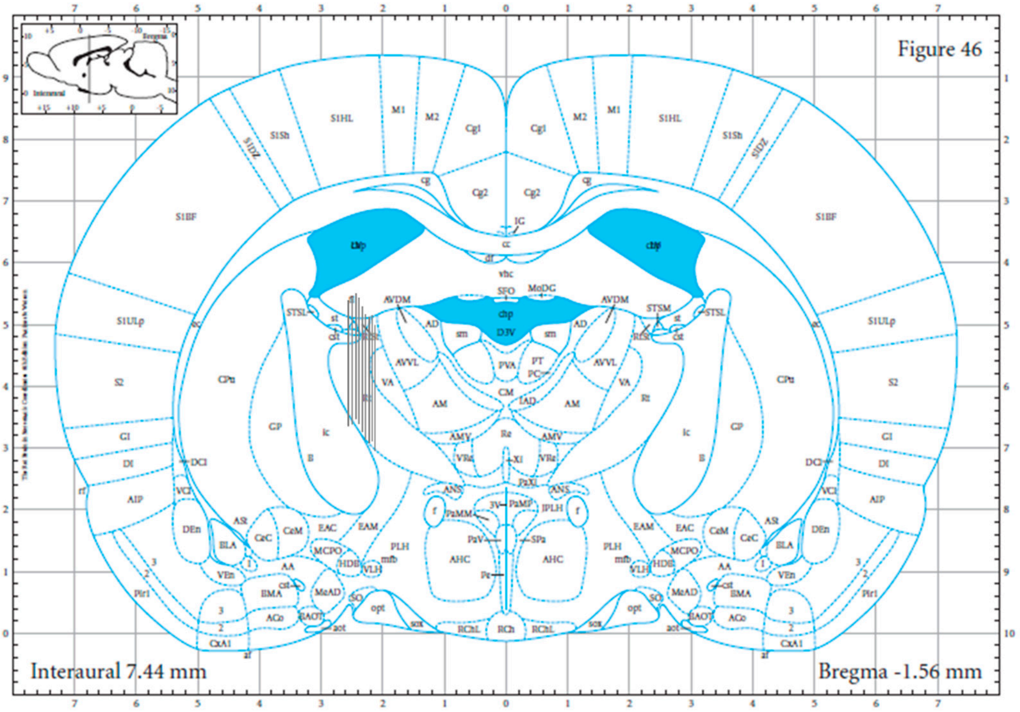

Figure S2.2.: Histological tracing of microdialysis probes in the reticular nucleus of thalamus

Supplementary Material S3

The extracellular basal levels of neurotransmitters in respective animal groups.

Table S3.1. Basal extracellular levels of dopamine (DA), serotonin (5-HT), glutamate and GABA in the rat frontal cortex and the reticular nucleus of thalamus before administration of psilocybin and ketamine.

**FRONTAL CORTEX**				
Treatment (mg/kg)	DA (pg/10 µL)	5-HT (pg/10 µL)	Glutamate (ng/10 µL)	GABA (pg/10 µL)
**Mean ± SEM (n)**				
control	1.99 ± 0.19 (14)	0.46 ± 0.05 (14)	1.27 ± 0.13 (14)	45.33 ± 4.5 (14)
Psilocybin 2	2.05 ± 0.16 (8)	0.48 ± 0.11 (6)	1.59 ± 0.19 (9)	47.0 ± 4.9 (9)
Psilocybin 10	2.11 ± 0.13 (9)	0.50 ± 0.08 (6)	1.21± 0.29 (8)	38.7 ± 4.6 (8)
Ketamine 10	1.82 ± 0.18 (12)	0.40 ± 0.03 (7)	1.02 ± 0.13 (6)	50.8 ± 7.2 (8)
**RETICULAR NUCLEUS OF THALAMUS**				
control	nd[1]	nd	3.83 ± 3.5 (6)	66.1 ± 6.1 (6)
Psilocybin 2	nd	nd	3.71 ± 0.41 (7)	64.9 ± 5.0 (6)
Psilocybin 10	nd	nd	3.27 ± 0.41 (6)	68.9 ± 13.1 (6)
Ketamine 10	nd	nd	4.50 ± 0.52 (8)	64.6 ± 7.8 (8)

[1] nd – not detected
